# Structural Homeostasis: Compensatory Adjustments of Dendritic Arbor Geometry in Response to Variations of Synaptic Input 

**DOI:** 10.1371/journal.pbio.0060260

**Published:** 2008-10-28

**Authors:** Marco Tripodi, Jan Felix Evers, Alex Mauss, Michael Bate, Matthias Landgraf

**Affiliations:** Department of Zoology, University of Cambridge, Cambridge, United Kingdom; Johns Hopkins University School of Medicine, United States of America

## Abstract

As the nervous system develops, there is an inherent variability in the connections formed between differentiating neurons. Despite this variability, neural circuits form that are functional and remarkably robust. One way in which neurons deal with variability in their inputs is through compensatory, homeostatic changes in their electrical properties. Here, we show that neurons also make compensatory adjustments to their structure. We analysed the development of dendrites on an identified central neuron (aCC) in the late *Drosophila* embryo at the stage when it receives its first connections and first becomes electrically active. At the same time, we charted the distribution of presynaptic sites on the developing postsynaptic arbor. Genetic manipulations of the presynaptic partners demonstrate that the postsynaptic dendritic arbor adjusts its growth to compensate for changes in the activity and density of synaptic sites. Blocking the synthesis or evoked release of presynaptic neurotransmitter results in greater dendritic extension. Conversely, an increase in the density of presynaptic release sites induces a reduction in the extent of the dendritic arbor. These growth adjustments occur locally in the arbor and are the result of the promotion or inhibition of growth of neurites in the proximity of presynaptic sites. We provide evidence that suggest a role for the postsynaptic activity state of protein kinase A in mediating this structural adjustment, which modifies dendritic growth in response to synaptic activity. These findings suggest that the dendritic arbor, at least during early stages of connectivity, behaves as a homeostatic device that adjusts its size and geometry to the level and the distribution of input received. The growing arbor thus counterbalances naturally occurring variations in synaptic density and activity so as to ensure that an appropriate level of input is achieved.

## Author Summary

As the nervous system develops, an intricate web of connections forms between nerve cells, leading to the assembly of signalling networks that are capable of complex computations. However, the number and strength of connections formed between nerve cells varies. We ask how nerve cells deal with this variability so that the circuits they form are nicely matched to the functions they perform. Nerve cells are known to adjust their sensitivity to compensate for changes in the strengths of inputs they receive from other cells. In this study, we have identified a structural counterpart to this compensatory mechanism, and find that developing nerve cells respond to variation in the number of connections they receive by adjusting the size of their receiving structures (known as dendrites). Working with the same nerve cell in different embryos, we show that this cell reduces the size of its dendrites as the number of connections increases while allowing its dendrites to grow more extensively if inputs are reduced. These findings suggest that, at least during the early stages of wiring the nervous system, nerve cells regulate the growth of their dendrites, to compensate for variability and attain an optimal number of connections.

## Abstract 3

Structural homeostasis is defined as follows: developing neurons modify the growth of their dendrites to compensate for changes in synaptic density. This structural adjustment is mediated, at least in part, by postsynaptic PKA signalling.

## Introduction

It is known that synapse formation on central neurons is subject to some degree of variability. This variability includes the positioning of synapses, their numbers, density, and strength. Despite the stochastic nature of the input that neurons receive, in normal circumstances, neural circuits are able to cope with the intrinsic variability of their connectivity and perform their function [1]. The robustness of these systems is a remarkable property. However, we have relatively little knowledge about how it is achieved. The work that we present here is intended to gain new insights into how neurons compensate for changes in the level, distribution, and density of the presynaptic input they receive. One attractive hypothesis is that neurons might actively modify the extent of their dendritic arbors to compensate for naturally occurring variations in synaptic input.

The relationship between synaptic input and dendritic development has been extensively investigated [2]. However, there is no clear agreement about the role of synaptic input in controlling arbor growth. In some cases, the arrival of synaptic input has been shown to induce an increase in postsynaptic dendritic elaboration. In these instances, the synaptic input would act as a trophic factor [3–5]. Other authors, however, have reached different conclusions [6–11], suggesting that the arrival of presynaptic input could act as a stop-growing or stabilization signal for the postsynaptic dendritic arbor. Comparing these different studies is difficult because many of them have been carried out in different systems and at different developmental stages. In addition, interpretations of the data are complicated by the fact that the results summarize the average effect of synaptic input on a more or less heterogeneous cohort of neurons rather than identified postsynaptic neurons.

One way of analyzing the role of presynaptic input in shaping the postsynaptic dendritic arbor would be to focus repeatedly, and under different experimental conditions, on the same developing neuron. At the same time, since synaptic contacts are likely to regulate dendritic growth, it would be important to be able to visualize not only the dendrites, but also the presynaptic contacts on them [12,13].

For these reasons, we decided to investigate the effect of synaptic activity and synaptic distribution on the growing dendritic arbor of a single identifiable neuron in the *Drosophila* embryo, namely the aCC motor neuron. This system, in comparison to vertebrate models, has the advantage of relying on the phenotypic analysis of a well-defined postsynaptic cell for which the developmental stage can be accurately determined, the synaptic input quantified, and whose presynaptic partners can be genetically manipulated.

Although a number of studies have focused on the genetic control of dendritic arbor development, the role of presynaptic activity in regulating the growth and branching of postsynaptic partner dendrites in *Drosophila* remains a largely unexplored question. Studies of the adult *Drosophila* visual system, however, suggest that visual activity regulates the volume of presynaptic terminals [14], but does not impinge on postsynaptic dendritic arbors [15].

The development of the electrical properties of the aCC motor neuron has been characterised in detail [16]. It is therefore possible to correlate the development of the dendritic arbor morphology with the acquisition of electrical characteristics. The only known excitatory inputs that the aCC motor neuron receives during embryogenesis are cholinergic [17], and the set of presynaptic cholinergic neurons can be manipulated genetically using the Gal4/UAS system [18,19]. We used this system to investigate in detail how the dendritic morphology of a single neuron changes as it develops, when the activity level and/or the density of its synaptic input are altered.

Our results indicate that the development of the dendritic arbor is tightly regulated by the synaptic input received. To our knowledge, this is the first evidence in *Drosophila* of dendritic remodelling in response to presynaptic activity. We show that abolishing presynaptic neurotransmitter synthesis or release results in an increase in dendritic complexity and in an increase of dendritic extension in a window of time that coincides with the onset of the first recordable excitatory postsynaptic events [16]. On the other hand, an increase in presynaptic density induces the opposite effect, resulting in a reduced dendritic arbor compared to controls.

The dendritic rearrangements that we observe in the course of these manipulations appear to occur locally, affecting branches in the proximity of synaptic sites. Through a survey of candidate genes, we were able to identify protein kinase A (PKA) signalling in the postsynaptic cell as the molecular pathway that mediates these activity-dependent dendritic rearrangements. These findings suggest that the dendritic arbor, at least during the initial stage of connectivity, behaves as a homeostatic structure that adjusts its geometry to compensate for changes in the activity or density of presynaptic release sites.

## Results

### Development of the aCC Dendritic Arbor

The aCC motor neuron can be identified in abdominal hemisegments of the *Drosophila* larval ventral nerve cord (VNC) [20]. The precision and the ease with which aCC can be located and manipulated make it a good model for studying dendritic development. In order to understand the dynamics of dendritic arbor growth, we followed the development of aCC over time, focusing on the developmental stages before and after connections first form on aCC, 14–18 h after egg laying (AEL) [16]. We injected the aCC neuron with Lucifer Yellow, and we then imaged it using confocal microscopy. We used custom-made software (see Materials and Methods for details) to reconstruct dendritic arbors and obtain quantitative data about their geometry [21–23].

**Figure 1:**
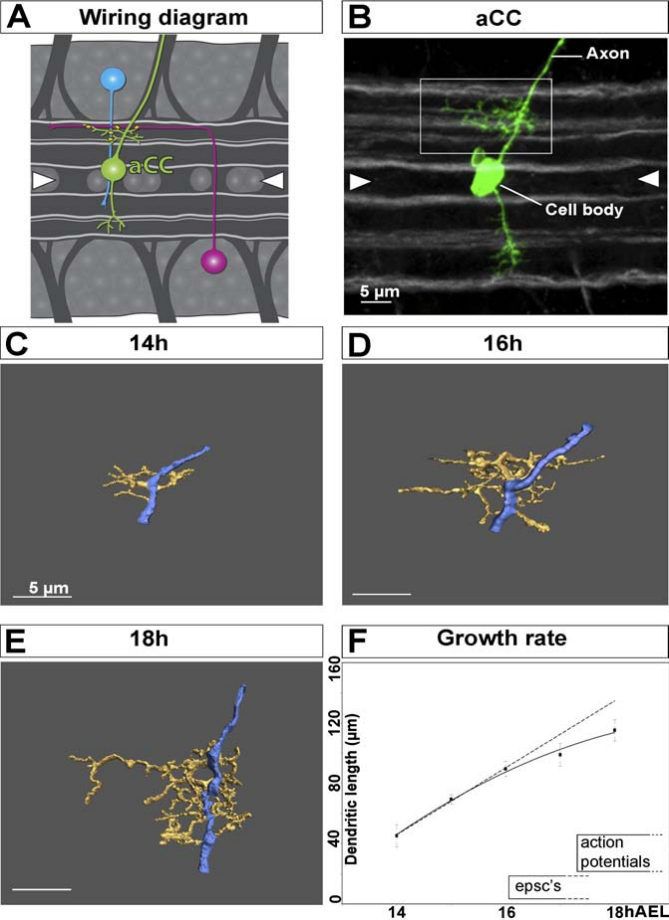
Development of the aCC Dendritic Arbor (A) Diagram of the ventral nerve cord (VNC) of a *Drosophila* embryo. The aCC is depicted in green, and two putative presynaptic cholinergic interneurons in blue and magenta. FasII-positive axon bundles are in grey, positioned in the lateral, intermediate, and medial neuropile. Note that this diagram illustrates a hypothetical pattern of connections. The actual number of interneurons presynaptic to motor neurons is currently unknown. (B) A 3-D projection of a confocal stack of an 18-h aCC (pseudocoloured green) in the context of the FasII-positive fascicles (grey). The box outlines the main (ipsilateral) dendritic arbor shown in the reconstructions below (C–E). Scale bar indicates 5 μm. (C–E) Reconstructions of aCC ipsilateral dendritic arbors at 14 h, 16 h, and 18 h AEL, respectively. Reconstructions are based on confocal image stacks of aCC neurons labelled by intracellular Lucifer Yellow injections. The dendrites are pseudocoloured yellow, and the axon/primary neurite, from which dendrites grow out, blue. For improved clarity, the cell body, located at the lower end of the axon, has been omitted from the reconstructions. Scale bar indicates 5 μm. (F) Growth curve of the aCC dendritic arbor: the *y*-axis indicates total tree length in μm; the *x*-axis indicates developmental stage in hours after egg laying (AEL). The dotted line shows a regression analysis calculated exclusively from data of early stages 14–16 h AEL. The resultant *R* ^2^ value is plotted above. The graph for 17 and 18 h AEL is an extrapolation at the growth rate calculated for earlier stages 14–16 h AEL. Times of onset of electrical properties of aCC are indicated [16]. Error bars indicate SEM. Anterior is left, and arrowheads indicate the ventral midline.

From 14–16 h AEL, the length of the dendritic arbor increases linearly with an average growth rate of 23 ± 2 μm per hour (tree length at 14 h: 45 ± 7 μm, *n* = 8; 15 h: 69 ± 3 μm, *n* = 9; and 16 h: 90 ± 5 μm, *n* = 11) (Figure 1C–1F). Between 16 and 18 h AEL, growth diverges from a linear increase as the average extension rate decreases by about 43% (13 ± 4 μm per hour; tree length at 17 h: 99 ± 8 μm, *n* = 8; and 18 h: 115 ± 7 μm, *n* = 13) (Figure 1D). Interestingly, 16 h AEL is the first stage at which evoked postsynaptic currents (EPSCs) can be recorded from aCC [16]. It seemed therefore possible that the onset of electrical activity might be the cause of the reduced rate of dendritic growth as of 16 h AEL, and we decided to investigate this further.

### A lack of Presynaptic Neurotransmitter Synthesis Induces Overgrowth of the Postsynaptic Dendritic Arbor

**Figure 2:**
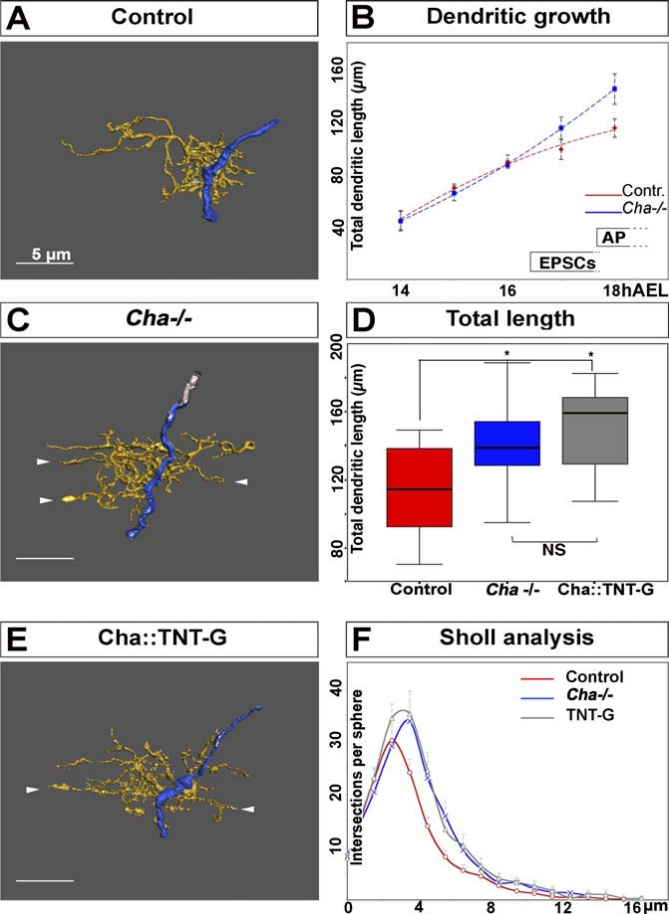
Lack of Presynaptic Neurotransmitter Induces Overgrowth of the Postsynaptic aCC Dendritic Arbor (A, C, and E) Reconstructions from confocal image stacks of representative aCC arbors at 18 h AEL. (A) control, (B) *Cha* mutants, and (C) tetanus toxin expressed in presynaptic neurons. Arrowheads in (C) and (E) indicate branches that show extra growth into neuropile territories not normally invaded. The dendrites are pseudocoloured yellow, and the axon blue. Anterior is left. Scale bar indicates 5 μm. (B) Growth curves of aCC dendritic trees in control (red) and *Cha* mutant animals (blue): the *y*-axis indicates total tree length in μm; the *x*-axis indicates developmental stage in hours AEL. AP, action potentials elicited in aCC by presynaptic inputs; NS indicates *p* > 0.05. (D) Quantification of dendritic tree length at 18 h AEL in control (red), *Cha* mutant (blue), and *Cha::TNT-G* animals (grey). Box-plots show the median of the distribution (middle line), the 75th percentile (upper limit of box), and 25th percentile (lower limit of box). Whiskers indicate the highest and lowest value of each experimental group. Significance was assessed by unpaired, two-tailed *t*-test. A single asterisk (*) indicates *p* < 0.05. (F) Sholl analysis for control neurons (red), *Cha* mutant (blue), and *Cha::TNT-G* (grey) animals. The axon was defined as origin. The *y*-axis indicates number of segment intersections; the *x*-axis indicates distance from the axon (origin) in micrometres. Error bars in B and F indicate SEM.

Since cholinergic neurons provide the only known excitatory input to *Drosophila* motor neurons in the embryo [16], we first analyzed the effect of the lack of neurotransmitter (synthesis) in the cholinergic neurons on the development of the aCC dendritic arbor. Acetyl choline is not synthesized in animals mutant for choline acetyl transferase (*Cha* ^l13^) [24], which are therefore immobile and unable to hatch. We charted the development of aCC dendritic arborisations in animals homozygous for the null mutation *Cha* ^l13^ [24]. In *Cha* mutant embryos, we find that the development of the aCC dendritic arbor proceeds normally until 16 h AEL (Figure 2B). However, in the interval between 16 to 18 h AEL, *Cha* mutants, unlike controls, fail to reduce the rate of dendritic growth (Figure 2B). As a result, at 18 h AEL, the extent of the aCC dendritic arbor is increased by about 26% in *Cha* mutants as compared to controls (from 115 ± 7 μm in control animals, *n* = 13; to 145 ± 14 μm in *Cha* mutants, *n* = 10, *p* = 0.03) (Figure 2B, compare Figure 2A with Figure 2C). We conclude that in the window of development, when in normal embryos acetyl choline–dependent excitation of aCC first begins [16] and the rate of dendritic growth declines, the absence of neurotransmitter in presynaptic neurons allows postsynaptic growth to continue linearly at an undiminished constant rate. These findings suggest that the aCC dendritic arbor reacts to the loss of synaptic input by increasing in size. A consequence of this increase in overall dendritic length is that it allows the dendritic arbor to explore a larger portion of the neuropile than in normal animals. In fact, we observe that in *Cha* mutants, the dendritic arbor of aCC extends into regions of the neuropile that are not normally invaded in control conditions (Figure 2C, arrowhead). This change in the dendritic distribution is also confirmed by the shift of the peak in the Sholl analysis (Figure 2F).

### Blocking Evoked Presynaptic Neurotransmitter Release Also Induces Overgrowth of the Postsynaptic Dendritic Arbor

We next investigated whether the dendritic phenotype observed in *Cha* mutants is indeed due to the silencing of the cholinergic population presynaptic to aCC or simply reflects a generic developmental defect caused by the absence of acetyl choline from such embryos. To this end, we specifically blocked evoked presynaptic release of neurotransmitter by targeting expression of tetanus toxin light chain (TNT-G) to cholinergic neurons using the GAL4/UAS system [17–19,25]. We found that the growth of the aCC dendritic arbor in these embryos did not differ from that observed in *Cha* mutants: the length of the aCC dendritic arbor is increased significantly with respect to controls (115 ± 7 μm) in embryos that are *Cha-GAL4; UAS-TNT-G* (from hereon abbreviated as *Cha::TNT-G*; 149 ± 10 μm, *n* = 10; *p* = 0.01) (Figure 2D, compare Figure 2A with Figure 2E), but is comparable to that seen in the *Cha* mutants (149 ± 10 μm in *Cha::TNT-G*, *n* = 10; *p* > 0.05) (Figure 2E). As is the case in *Cha* mutants, the arbor also explores a larger portion of the neuropile (Figure 2F).

Next, we sought to confirm that these effects on the aCC dendritic arbor are indeed due to blocking evoked neurotransmitter release in the subset of cholinergic neurons rather than a secondary consequence of general nerve cord development under these conditions. For instance, it has been reported that blocking evoked activity throughout the entire nervous system can impinge on the condensation of the VNC [26,27], which could lead to enlarged dendritic trees as we observe in the above manipulations. We therefore measured nerve cord condensation in *Cha::TNT-G* embryos 18 h AEL and found this to be normal (Figure S2).

Taken together, these data suggest that the overgrowth of the postsynaptic arbor that we observed in *Cha* mutants is a consequence of the absence of evoked presynaptic neurotransmitter release.

### Neurons Have Cell-Specific Programmes of Dendritic Growth and Branching on Which Presynaptic Input Impinges Quantitatively, but Not Qualitatively

The expansion of the aCC dendritic arbor, which results from an absence of evoked presynaptic neurotransmitter release, could be achieved in more than one way. One strategy would be an acceleration of the normal “programme of growth”: increasing the average growth rate of dendritic neurites while maintaining the normal frequency at which they branch. Alternatively, the same outcome of an enlarged dendritic tree could also be generated by changing the relationship between growth and branching, for instance, by increasing the rate at which dendritic neurites branch (and hence multiply) without altering their rate of extension. To understand how neurons adjust their dendritic arbors in response to alterations in input, we sought to distinguish between these alternative strategies. To do so, we asked what the relationship is between dendritic growth and branching. For the aCC neuron, there is a strong linear relation between dendritic tree length and number of branch points in control animals (*R* ^2^ = 0.75, *F*-value = 33, *p* < 0.05, *n* = 13). In animals from which presynaptic input had been removed (*Cha* mutants and *Cha::TNT-G*), this linear relation between tree length and branch points still holds (*Cha*: *R* ^2^ = 0.64, *F*-value = 11, *p* < 0.05; *Cha::TNT-G*: *R* ^2^ = 0.65, *n* = 10, *F*-value = 11, *p* < 0.05, *n* = 10). Moreover, aCC arbors in the three genotypes (control, *Cha* mutants, and *Cha::TNT-G*) are comparable in the average ratios between tree length and branch point number, and therefore have similar linear regression coefficients (*p* > 0.05, one-way ANOVA). This suggests that the ratio of dendritic length and number of branch points, i.e., the frequency of branching events per unit dendritic length, is an autonomous property of the aCC neuron that is not significantly altered by changes in presynaptic input. The extra dendritic growth observed in the absence of presynaptic input is therefore generated by the normal programme of (relationship between) growth and branching, which fails to decelerate between 16 and 18 h AEL under these conditions, as would be the case in the wild type.

**Figure 3:**
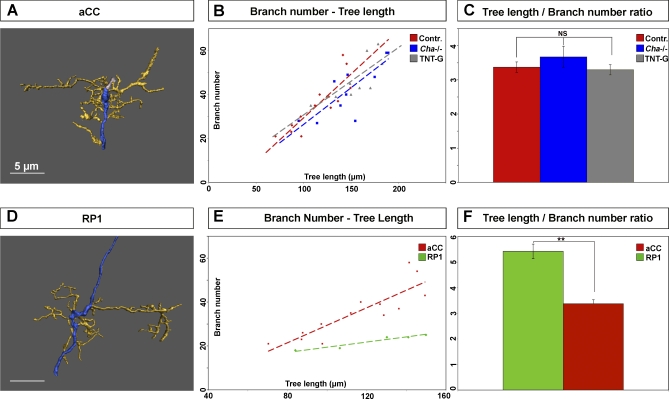
Neurons Have Type-Specific Patterns of Dendritic Growth and Branching That Are Modulated Quantitatively, but Not Qualitatively, by Presynaptic Input (A) Reconstruction of a representative aCC dendritic arbor at 18 h AEL. Scale bar indicates 5 μm. (B) Correlation analysis between dendritic tree length (*x*-axis, micrometres) and number of branches (*y*-axis) of aCC at 18 h AEL in different conditions: control (red), *Cha* mutants (blue), and *Cha::TNT-G* (grey). *R* ^2^ values are: controls (red), *R* ^2^ = 0.75, *F*-value = 33, *p* < 0.05, *n* = 13; *Cha* mutants (blue), *R* ^2^ = 0.64, *F*-value = 11, *p* < 0.05, *n* = 10; in Cha::TNT-G (yellow), *R* ^2^ = 0.65, *n* = 10, *F*-value = 11, *p* < 0.05, *n* = 10. Dashed lines indicate the slopes of the samples. (C) Bar plots (mean ± standard error of the mean [SEM]) of the ratio between total tree length (in micrometres) and number of branch points in control (red), *Cha* mutant (blue), and *Cha::TNT-G* animals (grey). (D) Reconstruction of a representative RP1 arbor at 18 h AEL. Scale bar indicates 5 μm. (E) Correlation analysis between dendritic tree length (*x*-axis, micrometres) and number of branches (*y*-axis) of aCC (red) and RP1 (green) at 18 h AEL. Both neurons have a linear relationship between tree length and branch number, *R* ^2^ = 0.93, *F*-value = 25, *p* < 0.05, *n* = 5. Dashed lines indicate the slope of the samples. (F) Ratios of tree length/branch number for aCC (red) and RP1 (green). The two differ significantly. Significance was assessed by unpaired, two-tailed *t*-test. Double asterisks (**) indicate *p* < 0.005, and NS indicates *p* > 0.05. Mean ± SEM. In all reconstructions, dendrites are pseudocoloured yellow and the axon blue.

It is conceivable that we observed a constant ratio between tree length and branch numbers under different experimental conditions because it reflects mechanical or cell biological constraints. If this were the case, then other neurons would be predicted to have the same ratio of tree length to branch number as aCC. To test this hypothesis, we analysed the arborisation pattern of another motor neuron, RP1. The RP1 motor neuron also shows a linear relation between tree length and branch points (*R* ^2^ = 0.93, *F*-value = 25, *p* < 0.05, *n* = 5). However, in the case of RP1, the ratio between tree length and branch points is significantly different from that observed for aCC (compare the two average tree-length/branch-point ratios in Figure 3F, *p* = 0.001, unpaired, two-tailed *t*-test, no equal variance assumed).

We conclude therefore that different neurons generate arbors with different intrinsic relationships between tree length and branch points. This ratio of dendritic growth and branching is probably determined by cell-autonomous differentiation programmes. It can be decelerated by presynaptic input, though not modified qualitatively.

### A Pattern of Cholinergic Synaptic Profiles Forms on the aCC Dendritic Arbor

**Figure 4:**
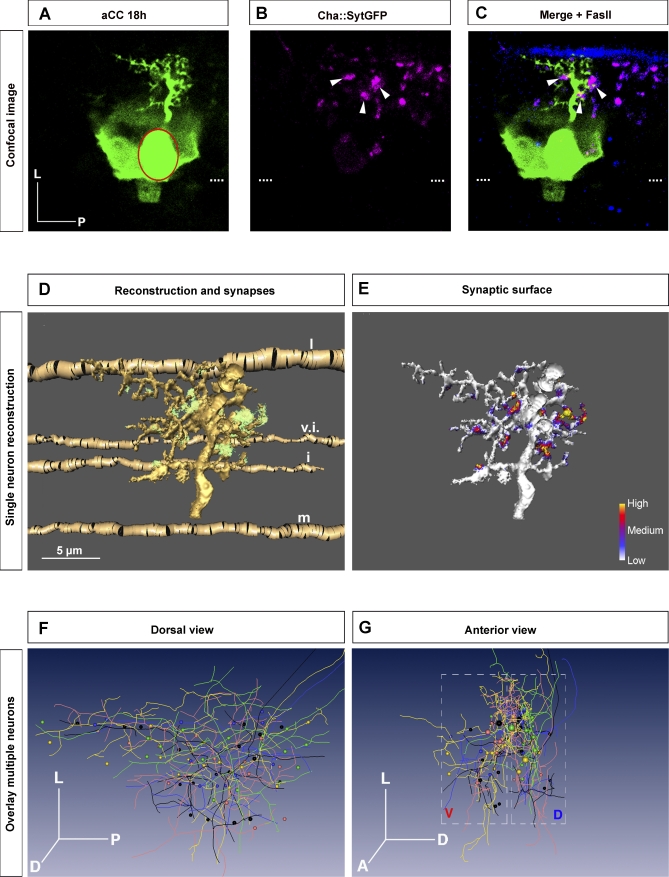
Synaptic Profile of the aCC Arbor at 18 h (A–C) Single confocal section of a representative Lucifer Yellow–labelled aCC neuron with dendritic arbor at 18 h AEL (green in [A and C]) and cholinergic presynaptic sites labelled with synaptotagmin-GFP (magenta in [B and C], arrowheads point to some contacts apposed to dendrites). The aCC cell body is outlined in (A) to distinguish it from the surrounding label of an overlying glial cell that was colabelled. (C) Overlay of the pseudocoloured images (A) and (B) and a third channel showing FasII-positive axons as landmarks (blue). Dashed lines indicate the ventral midline. (D) Reconstruction of an aCC arbor (yellow). Synaptic cholinergic contacts within a 300-nm radius of the reconstructed arbor are shown in green. Reconstructed FasII-positive axons (gold) are also shown: i = intermediate; l = lateral FasII fascicle; m = medial; v.i. = ventral intermediate. (E) Surface occupied by presynaptic sites plotted on the dendritic arbor reconstruction. The colour coding represents the relative intensity of synaptotagmin-GFP fluorescence staining within a 300-nm radius of the reconstructed arbor. (F and G) Overlay of reconstructions of five different aCC arbors at 18 h AEL. Optimal overlay was achieved by maximising the overlap of the FasII fascicles, which are independent, reliable landmarks in the neuropile; (F) dorsal, (G) anterior view. For greater clarity, skeletons of the reconstructions are shown and presynaptic sites are represented by spheres. Parts of the arbor: A, anterior; D, dorsal; L, lateral; P, posterior; V, ventral.

The growth and branching of dendrites is intimately linked to the formation of synaptic contacts on the dendritic tree [12,13]. We therefore sought to further our analysis by characterising the distribution of presynaptic sites formed on the aCC dendritic arbor, which is initially innervated, apparently exclusively, by cholinergic interneurons [16,17]. To do so, we expressed the presynaptic marker *UAS-synaptotagmin-GFP* [28] in the cholinergic neurons with *Cha-GAL4* [29] (Figure S1) and labelled aCC neurons by intracellular injections of Lucifer Yellow (Figure 4C). We confirmed that synaptotagmin-GFP does indeed label presynaptic sites by showing its colocalization with the endogenous presynaptic protein synapsin (Figure S1). In addition, we visualised as absolute reference points in the neuropile a set of axon bundles by anti-fasciclin II (FasII) staining (Figure 4C). We then used custom-made software (see Materials and Methods) to calculate from confocal image stacks of labelled specimens the areas of reconstructed aCC dendritic arbors closely apposed (within a 300-nm radius) by synaptotagmin-GFP puncta of cholinergic presynaptic terminals (Figure 4E) [21,22]. From hereon, we consider synaptotagmin-GFP puncta within a 300-nm radius of the dendritic arbor as indicative of presynaptic sites. This is an approximation as the imaging methods that we use cannot unambiguously identify actual or indeed active synapses.

We find that a reproducible pattern of presynaptic sites form on aCC dendrites by 18 h. This pattern can be observed by reconstructing aCC dendritic arbors and the presynaptic sites that they contact from multiple 18-h specimens when overlaid (with maximum overlap of FasII fascicles between specimens) (Figure 4F and 4G). There are two main regions where presynaptic sites contact the aCC arbor, which can be identified by absolute positions in the neuropile (Figure 4D) as well as by their relative positions within the dendritic tree (Figure 4E). Each of these main contact regions contains a variable number of synapses. The first group of synapses contacts the ventral aCC arbor (“V” in box in Figure 4G), the second group mainly contacts the dorsal anterior running dendritic branch (“D” in box in Figure 4G) located just medial to the lateral FasII fascicle (Figure 4D).

### Increased Density of Presynaptic Sites Induces a Compensatory Decrease in Postsynaptic Arbor Size

**Figure 5:**
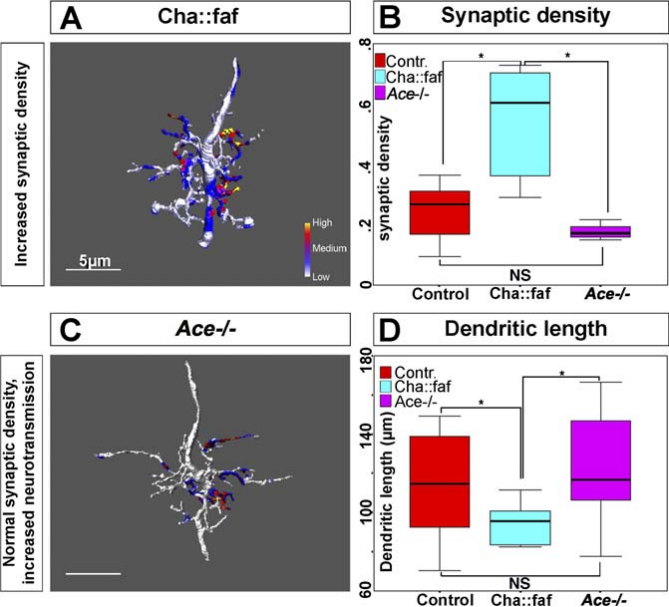
An Increase in Presynaptic Density, but Not Efficacy, Induces a Decrease of Postsynaptic Dendritic Arbor Extension (A) Reconstruction of a representative aCC arbor at 18 h AEL from a specimen in which *faf* was overexpressed in the presynaptic cholinergic neurons. Presynaptic sites on the arbor are indicated by coloration, with the colour code representing relative intensities of colocalised fluorescence signals. Scale bar indicates 5 μm. (B) Quantification of the synaptic density (dendritic surface occupied by synapses divided by total dendritic length) on the aCC arbor in control (red), *Cha::faf* (cyan), and *Ace* mutant animals (magenta). The synaptic density on the postsynaptic dendritic arbor is increased when *faf* is overexpressed presynaptically but not in *Ace* mutants (controls: 0.22 ± 0.03, *n* = 12; *Cha::faf* : 0.54 ± 0.07, *n* = 10; *Ace* mutants: 0.18 ± 0.01, *n* = 8). (C) Reconstruction of a representative aCC arbor of an *Ace* mutant animal. aCC arbors and the density of presynaptic cholinergic sites on these are comparable to controls. Scale bar indicates 5 μm. (D) Quantification of total dendritic tree length in control (red), *Cha::faf* (cyan), and *Ace* mutant animals (magenta). Presynaptic overexpression of *faf* induces reduced growth of the arbor (94 ± 4 μm, *n* = 10) as compared to controls (115 ± 7 μm, *n* = 13; *p* = 0.02). aCC arbors in *Ace* mutants are not significantly different from control animals (124 μm ± 14 μm, *n* = 8; *p* > 0.05). Box-plots show the median of the distribution (middle line), the 75th percentile (upper limit of box), and 25th percentile (lower limit of box). Whiskers indicate the highest and lowest value of each experimental group. Significance was assessed by unpaired, two-tailed *t*-test. A single asterisk (*) indicates *p* < 0.05; NS indicates *p* > 0.05.

Our findings show that the absence of synaptic input (e.g., in *Cha* mutant or *Cha::TNT-G* animals) induces an increase in dendritic arbor extension. This indicates that evoked neurotransmitter release acts as a stop-growing signal. This finding further suggests that the additional dendritic growth, which is induced by the absence of evoked neurotransmitter release, might be exploratory and represent a homeostatic mechanism that balances the growth of the dendritic arbor with the state of its connectivity. If this were the case, one would expect that increasing the density of synaptic sites (from which the stop-growing signal[s] originate) should induce the opposite effect, namely a reduction of dendritic arbor extension. To test this idea, we generated embryos in which the number of presynaptic sites on aCC was increased. At the *Drosophila* larval neuromuscular junction, the number of presynaptic release sites formed by the motor neuron on the postsynaptic target muscle can be increased considerably by neuronal (presynaptic) overexpression of the deubiquitination enzyme fat-facets (Faf) [30]. When we overexpressed *faf* in the cholinergic neurons (*Cha::faf*), we found that this manipulation caused an increase in the density of synapses formed on the aCC motor neuron. In order to obtain a quantitative readout of synaptic density, we measured the surface of cholinergic synapses on the aCC dendritic arbor and normalized this for arbor length (μm^2^ of synaptic surface divided by μm of dendritic length). Overexpressing *faf* in cholinergic neurons does indeed significantly increase the density of presynaptic sites on the aCC dendritic arbor at 18 h AEL (in control animals, the synaptic density is 0.22 ± 0.03 μm, *n* = 12; in *Cha*::*faf*, 0.54 ± 0.07 μm, *n* = 10; *p* = 0.003) (Figure 5B).

We next asked how the dendritic arbor responds to this increase in synaptic density. Under these conditions, the size of the aCC dendritic arbor is significantly reduced with respect to controls (in *Cha*::*faf* animals, the tree length is 94 ± 4 μm, *n* = 10; in controls, 115 ± 7 μm; *p* = 0.02, *n* = 12) (Figure 5A and 5D).

These results are consistent with the notion that a homeostatic mechanism operates to match the extent of dendritic growth to the availability of presynaptic sites. Abolishing synaptic activity, as shown in *Cha* mutants and when TNT-G is expressed presynaptically, induces an increase in dendritic arbor extension. On the other hand, an increase in the density of presynaptic sites induces the opposite effect, namely a decrease of arbor size.

### The Postsynaptic Dendritic Arbor Responds to an Increase in Density of Presynaptic Sites Rather Than to Elevated Neurotransmitter Release

The previous experiment shows that an increase in the density of presynaptic sites induces a reduction of the postsynaptic dendritic arbor extension. The cause of this effect could be that a higher density of presynaptic sites might lead to elevated amounts of neurotransmitter being released onto the arbor as a whole. In this case, the dendritic arbor would respond to overall levels of neurotransmitter across its entirety. Alternatively, dendritic size might be regulated locally, at each point of synaptic contact. In this second scenario, the critical point of dendritic growth regulation is the increase in the number of sites where neurotransmission can feed back on neurite extension. In order to distinguish between these alternatives, we analyzed the morphology of the aCC dendritic arbor and the density of its presynaptic sites in embryos mutant for acetyl choline esterase (*Ace*). In these mutants, presynaptically released acetyl choline fails to be degraded. As a result, overall levels of neurotransmitter are elevated, and there is an increase in cholinergic neurotransmission [31]. Our analysis shows that the number of presynaptic sites contacting aCC and their distribution are unchanged in *Ace* mutants (Figure 5B). We could also not detect any significant change in the extent and morphology of aCC dendritic arbors (124 μm ± 14 μm, *p* > 0.05, *n* = 8) (Figure 5C and 5D). These results show that increasing overall neurotransmitter levels, while leaving the density and distribution of presynaptic sites on the arbor unchanged, does not result in a growth adjustment of the dendritic arbor.

We therefore conclude that it is the density of sites where neurotransmitter release occurs that regulates dendritic tree size, rather than overall levels of neurotransmitter.

### Synaptic Sites Exert Local Inhibitory Effects on Dendritic Growth and Branching

**Figure 6:**
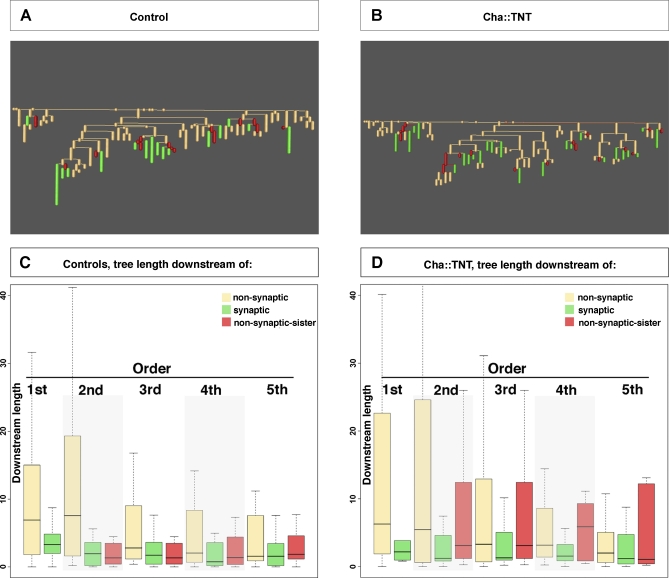
Synaptic Activity Locally Inhibits Postsynaptic Dendritic Arbor Extension (A and B) Dendritograms of a representative arbor from a control (A) and a *Cha::TNT-G* animal (B). The dendritograms depict the hierarchical branch orders of the arbors from the origin (i.e., axon, top branch) down. Neurites contacted by presynaptic sites are coloured in green, nonsynaptic sister branches in red. (C) Comparison of number of branch points downstream of other nonsynaptic neurites (non-syn) (beige); synaptic neurites (syn) (green); nonsynaptic sister neurites (non-syn-sist) (red), in control conditions. The *y*-axis indicates downstream tree length in micrometres; the *x*-axis indicates type of neurite and branch order. (D) Analysis of tree length downstream of the different types of neurites as for (C) in *Cha::TNT-G* animals. The *y*-axis indicates downstream tree length in micrometres; *x*-axis indicates type of neurite and branch order. Neurites are sorted by branch order, and results for the first five branch orders are shown. Box-plots show the median of the distribution (middle line), the 75th percentile (upper limit of box), and 25th percentile (lower limit of box). Whiskers extend 1.5 times the inter-quartile range; outliers are not plotted. Significance was assessed by the Wilcoxon rank-sum test. Double asterisks (**) indicate *p* < 0.005.

We have shown that the growth response of the postsynaptic dendritic arbor is regulated by the density of presynaptic sites. The implication of this finding is that dendritic growth is modulated locally at synaptic sites and that therefore the arrangement of synapses on the arbor might be important for shaping the dendritic geometry. To investigate this further, we asked whether, and to what extent, the inhibition of growth caused by presynaptic neurotransmitter release is spatially restricted to the particular neurite receiving the synaptic contact. We began our analysis with control animals, identifying all neurites of the dendritic arbor that are contacted by presynaptic sites. Next, for each branching order, we calculated and compared the dendritic length downstream of the following types of neurites: (1) “synaptic neurites” (those contacted by presynaptic sites); (2) “nonsynaptic sister neurites” (neurites originating form the same parental branch as a “synaptic neurite,” but not contacted by presynaptic sites); and (3) “other nonsynaptic neurites” in the arbor. We performed this analysis for the first five orders of the arbor. These comparisons show that both “synaptic” and “nonsynaptic sister neurites” have less dendritic length downstream than “other nonsynaptic neurites” of the same order (*p* << 0.005 for orders 1–3; *p* < 0.05 for order 4–5, Figure 6C). This result further suggests that dendritic growth appears to be regulated locally at sites of putative synaptic contacts.

To investigate the role of evoked neurotransmitter release in this local regulation of dendritic growth, we analysed aCC dendritic trees in animals in which evoked release was blocked by presynaptic expression of tetanus toxin (*Cha::TNT-G*). In both control and *Cha::TNT-G* animals, neurites contacted by presynaptic sites are comparable in length. The dendritic length downstream of these neurites is significantly shorter than that of “other nonsynaptic neurites” of the same order (in *Cha::TNT-G*, *p* << 0.005 for orders 1–3; *p* < 0.05 for order 4, *p* > 0.05 for order 5; Figure 6D). This suggests that dendritic extension is inhibited locally by the presence of presynaptic sites, though independent of their ability to release neurotransmitter.

However, when comparing the “nonsynaptic sister neurites between *Cha::TNT-G* animals and controls, we find that a block of evoked neurotransmission has an effect. The dendritic length downstream of nonsynaptic sister neurites is significantly longer in *Cha::TNT-G* animals than in controls (*p* << 0.005 for all orders; compare red box-plots in Figure 6C and 6D). In *Cha::TNT-G* animals, nonsynaptic sister neurites are also longer than synaptic neurites of the same order (*p* << 0.005 for all orders, compare red and green box-plots in Figure 6D). This indicates that the presence of presynaptic sites inhibits growth of neighbouring (sister) neurites in an activity-dependent manner.

In summary, these results highlight two interesting features. First, neurites contacted by presynaptic sites extend less than nonsynaptic neurites. This inhibitory effect on these dendritic segments is not dependent on evoked neurotransmitter release since it is present in both control and *Cha::TNT-G* animals. Second, another inhibitory effect of synaptic sites on dendritic growth exists, which extends to neighbouring (sister) neurites. Interestingly, this type of inhibition requires evoked neurotransmitter release, since this is absent in *Cha::TNT-G* animals.

### Dendritic Growth Is Inhibited by Contact with Presynaptic Terminals Independent of Neurotransmitter Release

**Figure 7:**
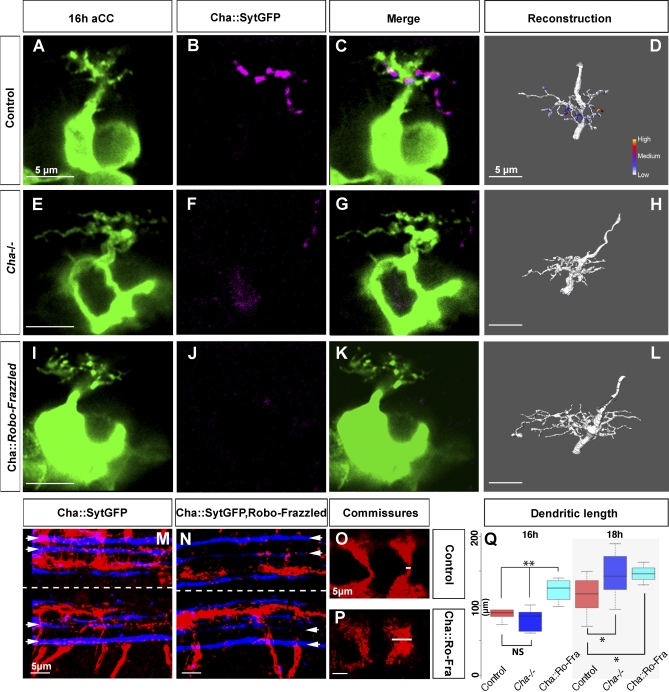
Presynaptic Sites Induce an Activity-Independent Local Inhibition of Postsynaptic Dendritic Growth (A–C) Single confocal sections of a Lucifer Yellow–labelled aCC at 16 h AEL (pseudocoloured green), cholinergic presynaptic sites (*Cha::SytGFP*, pseudocoloured magenta), and the overlay of the two channels, respectively. (D) Reconstruction of the aCC arbor shown in (A), areas of contact with the arbor colour coded to represent relative intensities of colocalised fluorescence signals. (E–H) As for (A–D), but for *Cha* mutants at 16 h AEL. (I–L) As for (A–D), but for animals in which the chimeric receptor Robo^ectodomain^-Frazzled^cytoplasmic^ (Robo-Frazzled) has been expressed in the cholinergic neurons. (M and N) Projections of the dorsal neuropile in control (M) and *Cha::Robo-Frazzled* (N) animals showing presynaptic cholinergic terminals (red), and FasII-positive fascicles (blue). In *Cha::Robo-Frazzled* (N) animals, terminals are shifted from lateral and intermediate (as demarcated by the lateral and intermediate FasII-positive fascicles [arrowheads]) to medial regions of the neuropile (the ventral midline is indicated by dashed lines). (O and P) Projections of cholinergic presynaptic sites in the posterior commissure. Two adjacent abdominal segments are shown. There is a marked increased width of the posterior commissure, indicated by white bar on ventral midline, in *Cha::Robo-Fra* (P) as compared to control (O) animals. (Q) Quantification of aCC total dendritic tree length at 16 h and 18 h AEL for controls (red, *Cha* mutant (blue), and *Cha::Robo-Frazzled* (cyan) animals. Box-plots show the median of the distribution (middle line), the 75th percentile (upper limit of box), and 25th percentile (lower limit of box). Whiskers indicate the highest and lowest value of each experimental group. Significance was assessed by unpaired, two-tailed *t*-test; double asterisks (**) indicate *p* < 0.005, and NS indicate *p* > 0.05. Scale bars indicate 5 μm.

These results suggest that the local inhibition of dendritic growth at the site where putative synapses form depends on mechanisms other than evoked release. This hypothesis predicts that the postsynaptic dendritic arbor would respond with overgrowth to the absence of contact with presynaptic partner terminals. To test this, we sought to prevent the normal formation of contacts between pre- and postsynaptic partners by shifting the presynaptic terminals of cholinergic neurons to a more medial portion of the neuropile. To do so, we expressed in the cholinergic cells (with *Cha-GAL4*) the chimeric Robo^ectodomain^-Frazzled^cytoplasmic^ guidance receptor, which has been shown to induce a strong medial shift of axons [32]. The expression of this chimeric receptor in the cholinergic population does indeed induce a shift of cholinergic synaptic terminals out of the lateral and into the medial neuropile (compare Figure 7M with 7N and Figure 7O with 7P).

So as to be able to differentiate clearly between contact- and activity-dependent effects, we focused on aCC arbors in 16-h-old specimens. At this stage, presynaptic sites can be detected on the aCC dendrites in positions resembling those of more mature arbors. However, these synapses are largely immature, as evoked synaptic currents are only just beginning to occur in aCC at 16 h AEL [16], and a lack of transmission at this stage fails to induce a postsynaptic response of dendritic overgrowth, as shown earlier (Figure 2B).

We find that shifting the presynaptic cholinergic terminals into the medial part of the neuropile results in a significant reduction in presynaptic sites on the aCC dendritic arbor (Figure 7L).

Next, we compared the extent of the dendritic arbor at 16 h in three different genotypes: control condition (*Cha::SytGFP*), *Cha* mutants (*Cha* ^−/−^ ; *Cha::SytGFP*), and in animals in which the Robo^ectodomain^-Frazzled^cytoplasmic^ chimeric receptor was expressed in the cholinergic population (*Cha::SytGFP::Robo-Frazzled*). As we have shown before, at 16 h, the extent of the aCC arbor in *Cha* mutants is comparable to that of control animals (Figure 7H and 7Q), demonstrating that up to this stage, dendritic growth is not obviously modulated by the presence or absence of neurotransmitter. However, displacing the presynaptic terminals causes a significant increase in the dendritic arborisation of aCC with respect to both control and *Cha* mutant animals: tree length in controls, 89.5 ± 5 μm, *n* = 11; in *Cha* mutants, 81 ± 6.5 μm, *n* = 8; in *Cha::Robo-Frazzled*, 123 ± 7 μm, *n* = 9; *p*(control, *Cha*) > 0.5; *p*(control, *Cha::Robo-Frazzled*) = 0.02; *p*(*Cha*, *Cha::Robo-Frazzled*) = 0.009 (Figure 7L–7Q). We find that this effect persists at least until 18 h AEL: tree length in controls, 115 ± 7 μm; in *Cha::Robo-Frazzled*, 146 ± 5 μm; in *Cha* mutants 145 ± 11 μm; *p*(control, *Cha::Robo-Frazzled*) = 0.009; *p*(*Cha*, *Cha::Robo-Frazzled*) > 0.05.

The comparison at 16 h between *Cha* mutants and animals with displaced presynaptic cholinergic terminals (*Cha::Robo^ectodomain^-Frazzled^cytoplasmic^*) indicates (1) that simple contact between pre- and postsynaptic partners has an inhibitory effect on the growth of the postsynaptic dendritic arbor, and (2) that this effect is not due to, and therefore independent of, neurotransmitter release. By 18 h AEL, the effect of displacing presynaptic terminals has become less pronounced and is comparable to the increase in dendritic tree size that results when neurotransmission is absent (e.g., in *Cha* mutants).

It has been suggested that alterations to the Slit distribution affect dendritic development [33]. The set of FasII fascicles has been used as a standard indicator for Slit signalling in the VNC [34,35], and few *Cha-GAL4* positive axons contribute to this set (unpublished data). Quantifications show that the distribution of the FasII fascicles is unaltered between *Cha::Robo-Frazzled* and control animals (Figure S3). This suggests that the Slit distribution is not affected by our manipulations and that the dendritic phenotype that we observe is indeed the result of the shift of the presynaptic partners.

On the basis of these observations, we speculate that the local inhibition of growth evident in neurites with presynaptic sites is contact mediated. In contrast, the inhibitory effect that extends to the nonsynaptic sister neurites is likely to be transmitter dependent, since this inhibition is lost in the absence of evoked presynaptic release (*Cha::TNT-G*).

### Presynaptic Release of Neurotransmitter Signals through Postsynaptic Protein Kinase A Activation to Repress Dendritic Growth

We have shown that presynaptic sites exert an “extended” inhibitory effect on the growth of neighbouring (sister) neurites and that this inhibition requires presynaptic release of neurotransmitter. However, the nature of the postsynaptic signals that mediate this effect is unclear. The activation of a number of postsynaptic signalling cascades has been proposed as a response to presynaptic neurotransmitter release. The best-characterised ones rely on Ca^2+^ entry following synaptic excitation [36]. For aCC, alterations in synaptic input have been shown to induce rapid changes in its excitability that are mediated by cAMP-dependent phosphorylation of PKA [31]. We therefore asked whether PKA might also be involved in the regulation of dendritic growth in response to variations of presynaptic neurotransmitter release.

**Figure 8:**
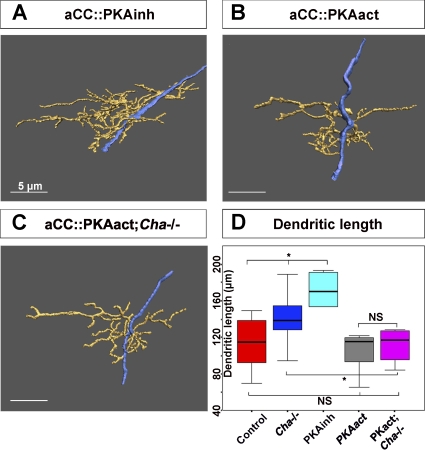
The Activity-Dependent Inhibition of Dendritic Growth Is Mediated by Protein Kinase A (A) Dendritic arbor reconstruction of a representative aCC neuron at 18 h AEL expressing a constitutive inhibitor of PKA activity (*PKAinh*). Postsynaptic expression of *PKAinh* leads to a significant increase in dendritic length (see [B and D] for comparison). (B) Reconstruction of a representative dendritic arbor of an aCC neuron at 18 h AEL expressing a constitutively active form of PKA (*PKAact*) does not affect the growth of the dendritic arbor, which is comparable to controls (see [A and D] for comparison). (C) As for (B), but in a *Cha* mutant background. Expression of *PKAact* in aCC can fully rescue the dendritic overgrowth phenotype of *Cha* mutants. (D) Comparison of aCC dendritic tree length at 18 h AEL in controls, 115 ± 7 μm, *n* = 13 (red); *Cha* mutants, 145 ± 14 μm, *n* = 10 (blue); upon expression of *PKAinh* in aCC, 167 ± 16 μm, *n* = 10 (cyan); upon expression of *PKAact* in aCC, 105 ± 9 μm, *n* = 9 (grey); in *Cha* mutants upon expression of *PKAact* in aCC, 112 ± 10 μm, *n* = 10 (magenta). Box-plots show the median of the distribution (middle line), the 75th percentile (upper limit of box), and 25th percentile (lower limit of box). Whiskers indicate the highest and lowest value of each experimental group. Significance was assessed by unpaired, two-tailed *t*-test; a single asterisk (*) indicates *p* < 0.05, and NS indicates *p* > 0.05. In all reconstructions, dendrites are pseudocoloured yellow and the axon blue. Expression of *PKAact* and *PKAinh* was targeted to aCC using the *RN2-GAL4* line [38]. Scale bars indicate 5 μm.

In order to test this hypothesis, we first targeted expression of a dominant-negative regulatory subunit of PKA, *PKAinh* [37], to aCC using the *RN2-GAL4* line [38]. This manipulation induces dendritic overgrowth similar to that observed in *Cha* mutants (tree length: 167.5 ± 16.1 μm, *p* (control) = 0.03, *n* = 10) (Figure 8A and 8D). Next, we overexpressed a constitutively active form of PKA, *PKAact* [37]. Interestingly, this has no effect on the dendritic phenotype when overexpressed in aCC in otherwise wild-type animals (tree length: 105 μm ± 9 μm, *p* > 0.05, *n* = 8) (Figure 8B and 8D). Since PKA is probably downstream of several signalling pathways, the observation that overexpressing the dominant-negative form induces dendritic overgrowth does not in itself prove that PKA is acting downstream of presynaptic neurotransmitter release. To provide evidence on this point, we asked whether postsynaptic expression of the constitutively active form of PKA could rescue the dendritic overgrowth phenotype induced by the absence of neurotransmitter in *Cha* mutants. We find that *PKAact* overexpression in aCC does indeed fully rescue the postsynaptic overgrowth observed in *Cha* mutants (tree length: 112 μm ± 10, *p* = 0.03, *n* = 10) (Figure 8C and 8D). This observation together with the fact that the overexpression of *PKAact* in otherwise wild-type animals does not induce a dendritic phenotype supports the idea that PKA signalling is part of the postsynaptic mediator of the dendritic morphological adjustments induced by presynaptic neurotransmitter release.

### Naturally Occurring Variations in the Density of Cholinergic Terminals in the Motor Neuropile Correlate with Variations in aCC Dendritic Arbor Extension

We have used various genetic approaches to manipulate the synaptic input received by the aCC motor neuron. We have shown that in response, the aCC dendritic arbor modifies its dendritic morphology based on the density of active synaptic contacts. These findings suggest that in normal conditions, naturally occurring variations in the density of presynaptic cholinergic terminals in the motor neuropile might also be counterbalanced by homeostatic changes in the geometry of the aCC dendritic arbor.

**Figure 9:**
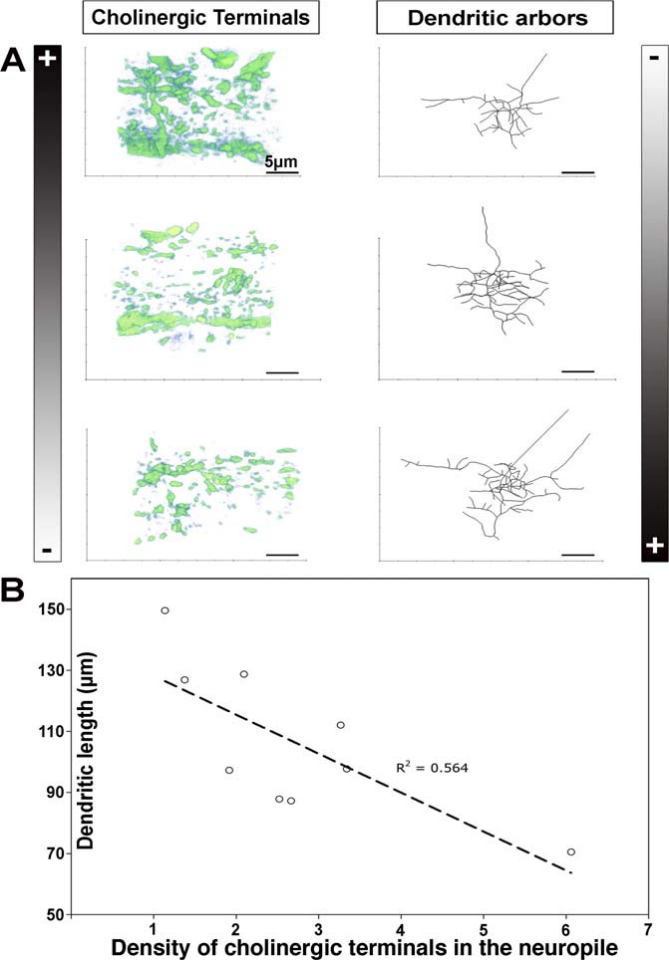
Correlation between Naturally Occurring Variations in the Density of Presynaptic Cholinergic Terminals and Postsynaptic Dendritic Arbor Extension (A) The left column shows 3-D projections of cholinergic presynaptic profiles (*Cha::Syt-GFP*, anti-GFP staining) in the motor neuropile volumes (14.4 μm × 14.4 μm × 6 μm deep) containing the 18 h AEL aCC arbors depicted on the right. The presynaptic cholinergic profiles and their quantification were generated with the “Analyze Particles” ImageJ Plugin. aCC arbors are depicted as skeletons of reconstructions. (B) Correlation analysis of aCC dendritic tree length and the density of staining of the presynaptic cholinergic terminals in the motor neuropile. The dashed line indicates the linear best fit to the data points. The *x*-axis indicates synaptic density as the percentage of the neuropile volume containing staining for cholinergic presynaptic sites; the *y*-axis indicates total aCC dendritic length in micrometres in the same animal as the corresponding *x*-value; *R* ^2^ = 0.6; *F*-value = 9; *p* = 0.02, *n* = 12. Scale bars indicate 5 μm.

We put this prediction to the test. Using multiple control specimens, we first asked whether the density of presynaptic cholinergic terminals in the motor neuropile varies between control specimens. We find that it does (Figure 9A, left column). We then quantified the length of aCC dendritic trees in these specimens (Figure 9A, right column) and asked whether naturally occurring variations in the density of cholinergic terminals in the motor neuropile correlate with aCC dendritic arbor size. We find that there is a significant linear correlation between the density of cholinergic terminals in the motor neuropile and the extent of the aCC dendritic arbor (*R* ^2^ = 0.6; *F* value = 9; *p* = 0.02, *n* = 9) (Figure 9B). A higher density of cholinergic terminals corresponds to a shorter postsynaptic dendritic arbor and vice versa (Figure 9A).

Overall, these findings support the model we propose, namely that the aCC dendritic arbor behaves as a homeostatic device that adjusts its geometry to compensate for naturally occurring variations in the availability of cholinergic terminals.

## Discussion

**Figure 10:**
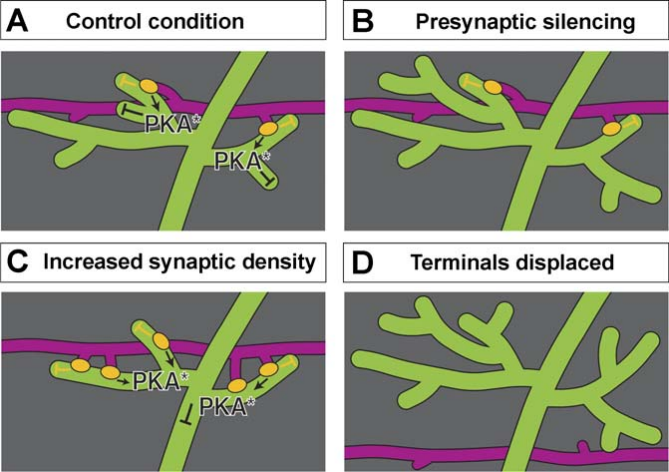
Model of the Role of Presynaptic Partners in Shaping Postsynaptic Dendritic Arbors Cartoons of postsynaptic dendrites (green), presynaptic partner neurons (magenta), and synaptic sites (yellow). (A) In the wild type, (synaptic) contacts by presynaptic terminals act locally to restrict growth of the contacted postsynaptic neurites. This effect is independent of activity. Synaptic input exerts a second “extended” inhibitory effect on neighbouring nonsynaptic sister neurites. This second effect is activity dependent and mediated through PKA signalling. (B) In the absence of presynaptic neurotransmitter release (e.g., in *Cha* mutant and *Cha::TNT-G* animals), there is no activity-dependent, PKA-mediated “extended” inhibition. As a result, nonsynaptic neurites adjacent to presynaptic sites can continue to grow. In contrast, growth of synaptic neurites remains repressed by local contact-meditated, activity-independent mechanisms. Regions of the dendritic arbor that lack presynaptic input explore the neuropile further. (C) Increasing the density of active presynaptic terminals (e.g., in *Cha::faf* animals) leads to less extended and elaborate postsynaptic dendritic arbors. Active presynaptic sites are in immediate reach of dendrites and, after establishing contact, exert local contact-mediated and “extended” activity-dependent inhibition of further dendritic growth. (D) Displacement of presynaptic partner terminals (e.g., in *Cha::Robo-Frazzled* animals) eliminates contact with the postsynaptic arbor and thereby also relieves both local and “extended” inhibition. As a result, the postsynaptic arbor responds with extended growth.

This study shows that the postsynaptic dendritic arbor computes the synaptic input that it receives and adjusts its arborisation to compensate for variations in the activity and/or the density of the presynaptic input. Abolishing synaptic input induces a compensatory overgrowth of the postsynaptic dendritic arbor (Figure 10B). On the other hand, increasing the density of presynaptic sites produces the opposite effect, reducing the growth of the postsynaptic dendritic arbor (Figure 10C).

These growth adjustments by the aCC arbor appear to operate sequentially at two levels. In the first instance, the dendritic arbor determines the presence or absence of presynaptic partners. This event is independent of presynaptic activity. It is also a local event that appears to affect primarily neurites receiving presynaptic sites, which act as a local stop-growing signal (Figure 10A). The second step depends on the activity of the synapse. In normal conditions, presynaptic sites are able to inhibit growth, both in synaptic neurites as well as neighbouring nonsynaptic sister neurites (Figure 10A). Interestingly, the dendritic arbor does not measure the efficacy of synapses (since there is no significant change in arbor size in *Ace* mutants as compared to wild type; Figure 5D), but simply determines whether a synapse is active or not. However, we cannot exclude the possibility of there being a compensatory response to reduced efficacy. This phase of activity-dependent inhibition of dendritic growth is mediated by postsynaptic activation of PKA.

### Presynaptic Activity Modulates Dendritic Arbor Growth

The morphology of dendrites is likely to be an important determinant of the connectivity state of a nervous system. It is not by chance that even in complex nervous systems, one of the most distinctive feature of different classes of neurons is probably the morphology of their dendritic arbors [39]. Indeed, the anatomical discrimination of different classes of neurons, based on their dendritic morphology, has often anticipated and predicted molecular, electrophysiological, and computational differences [40–46].

Therefore, an appreciation of the logic governing the assembly of a nervous system must include an understanding of how dendritic morphology is acquired. Although many of the distinctive features that differentiate the dendritic morphologies of different classes of neurons are likely to be cell-autonomously and genetically determined [47], the effect of partner-derived cues, as we show here, can have a substantial impact in modulating these features. We initiated our investigation by analysing the effect of presynaptic transmission in shaping the morphology of the postsynaptic dendritic arbor. Previous studies on this issue have not reached a clear agreement on the role of activity in regulating dendritic growth [2,3]. Even though in some instances it has been reported that activity has no effect on regulating arbor growth [48], in the majority of studies, activity emerges clearly as an essential modulator of dendritic remodelling. The main issue has been whether the incoming presynaptic input acts as a trophic factor that promotes arbor growth or whether it delivers a stop-growing signal. Unfortunately, with a few notable exceptions [5,6], many of these studies were carried out in different animals, in different neural populations, at different developmental stages, and by using different experimental approaches (genetic manipulation or pharmacological treatments), thus making it difficult to find a common theme in the results. As noted elsewhere [2,3], one major source of variation that could explain the differences in the results of previous studies is likely to be the developmental stage at which manipulations were applied. For instance, a single class of tectal neurons in *Xenopus* tadpoles appears to respond in opposite ways to presynaptic input at different developmental stages. In immature neurons, presynaptic input acts as a growth-promoting signal [5], whereas in mature neurons, it acts as a stop-growing or stabilization signal [6]. In this study, we find no evidence for a growth-promoting effect of synaptic activity. Instead, we show that synaptic input inhibits dendritic growth starting from the earliest stages at which neurotransmission occurs (16–18 h AEL). Altering activity before the onset of evoked synaptic transmission causes no dendritic phenotype (14–16 h AEL). It appears that developing *Drosophila* embryonic motor neurons behave like mature tectal neurons in *Xenopus*.

As very elegantly shown by Wu and Cline [6], the difference in the effect obtained at different stages in *Xenopus* tectal neurons nicely correlates with a change in their molecular characteristics. At later stages, when synaptic input acts as a stop-growing signal, the tectal neurons in *Xenopus* have acquired the ability to respond to local calcium increases via the activation of a calcium-dependent protein kinase CamKII [6]. In *Drosophila*, aCC appears be sensitive to the activation state of a different protein kinase, PKA (which is also regulated, albeit indirectly, by intracellular calcium levels) throughout the interval of development that we have studied.

### Local Effects of Synaptic Input and Presynaptic Sites on Neurite Extension

A great deal of attention has been given to global changes in postsynaptic activity and how these changes might regulate global dendritic growth. However, although this is an extremely interesting question, it is difficult to imagine how global variations in the state of activation of the whole dendritic arbor or the soma might contribute to fine-tuning the morphology of dendritic arbors. Far more compelling would be a system that was able to calculate and respond to local changes in activity levels. It is well known that calcium levels can be altered locally at the synaptic site following synaptic input [36,49–51]. It is also known that changes in dendritic levels of calcium can induce dramatic changes in dendritic morphology [49,51]. Nevertheless, there have been few investigations of how dendritic morphology might be altered locally by synaptic activity [12,52].

Because the system we use allows us to study a single identified postsynaptic neuron whose presynaptic input can be altered, we have been able to begin to address the issue of local versus global changes of dendritic morphology induced by synaptic activity. Analyzing the branching pattern of the dendritic arbor with respect to the position of its synaptic inputs highlights some interesting and unexpected features. By simply looking in wild-type animals, it is clear that neurites bearing presynaptic sites branch less than nonsynaptic neurites of the same arbor. Through experimental manipulation, we showed that this local inhibition of the growth and branching of synaptic neurites appears to be mediated by contact between pre- and postsynaptic partners and does not require evoked transmission. The lack of this contact-dependent inhibition of dendritic growth is already apparent when the terminals normally presynaptic to aCC are mistargeted before they can form functional synapses at 16 h AEL (Figure 10D). Moreover, this effect cannot be attributed to an interference with transmission, since *Cha* mutants of the same stage (16 h AEL) do not show this dendritic overgrowth phenotype. Loss of neurotransmitter release, on the other hand, can also induce an overgrowth of the postsynaptic dendritic arbor, though only after synapses would have been active for 2 h in normal conditions (i.e., 18 h AEL). We find that this neurotransmitter-dependent overgrowth is due to increased extension of nonsynaptic segments that are immediately adjacent to neurites receiving presynaptic sites. Thus, neurotransmitter release at the synaptic sites acts on the nonsynaptic sister neurites to inhibit their extension.

We conclude that the geometry of the dendritic tree is regulated by two partner-dependent mechanisms. First, contact with presynaptic terminals locally inhibits dendritic growth and branching in an activity-independent fashion. A second inhibitory effect requires evoked presynaptic neurotransmitter release. It extends from these sites to the immediate proximity, affecting nonsynaptic sister branches. This second “neighbourhood effect” could be mediated by local increases in dendritic calcium levels.

### PKA Is a Mediator of Presynaptic Neurotransmitter Release

Neurotransmission-dependent variations in intracellular calcium levels are an attractive mechanism that might implement activity-dependent local rearrangement of the dendritic arbor geometry. We therefore investigated the role of the protein kinase PKA in dendritic remodelling, as its activity is regulated directly or indirectly by calcium levels. PKA is activated by increases in the intracellular levels of cAMP. It has been shown that levels of cAMP are finely regulated by intracellular levels of calcium, which in turn respond to levels of presynaptic neurotransmitter release [49,51,53].

PKA signalling in postsynaptic cells has been shown to mediate homeostatic responses. For instance, at the neuromuscular junction, postsynaptic PKA signalling modulates quantal size [37], while centrally, it mediates homeostatic change in the electrical excitability of aCC neurons following alterations to presynaptic input [31]. In this study, we show that PKA activity is also a potent modulator of dendritic morphology. We show that PKA signalling is probably downstream of presynaptic transmission mediating the inhibition of dendritic growth. Overexpression of a constitutively active form of PKA (*PKAact*) is able to rescue the dendritic overgrowth phenotype that ensues in the absence of presynaptic transmission in *Cha* mutants. However, overexpression of *PKAact* in aCC in control animals has no measurable effect on dendritic development, suggesting that PKA signalling operates to saturation under normal levels of synaptic input. Although neurotransmission is clearly one signal that regulates dendritic development through downstream PKA signalling, it is not necessarily the only one.

### Structural Homeostasis

The term *homeostasis* has classically been used to refer to compensatory variations in the electrical properties of neurons that tend to counterbalance changes in their synaptic input [37,54–59]. We would like to propose here that neurons might combine the homeostatic regulation of electrical properties with compensatory structural adjustments of their dendritic geometry. We argue that variations in the morphology of aCC dendritic arbors represent such compensatory adjustment of dendritic growth and branching.

Our experimental observations indicate that eliminating synaptic input induces a compensatory dendritic overgrowth in the postsynaptic neuron, whereas an increase in the density of active synapses induces the opposite effect. Interestingly, increasing the overall level of neurotransmitter released at presynaptic terminals without altering the density of presynaptic sites on the arbor does not induce compensatory adjustments of the postsynaptic arborisation. This suggests that the compensatory changes in dendritic arbor morphology that we observe act to compensate for variations in the density of active synaptic release sites rather than variations in the global state of dendritic or neuronal depolarization. This is in agreement with our observations that changes in dendritic morphology in response to changes in presynaptic input or synaptic density appear to be implemented locally rather than across the entire arbor. This structural homeostasis, therefore, seems to work on a local scale, allowing particular regions of the dendritic arbor to compensate for variation in their inputs while leaving other regions of the arbor substantially unchanged. This could be an effective mechanism for neurons that use distinct regions of their dendritic arbor to independently compute different inputs [60–62].

It will be interesting to understand whether electrical homeostasis and what we propose to be a structural homeostasis operate in concert to shape the postsynaptic response, or whether one or the other is preferentially deployed depending on the circumstances. At the moment, we are unable to answer this question, and more experiments are required.

Nonetheless, one can begin to envisage how PKA signalling in the context of electrical homeostasis might be integrated with the structural homeostasis that we have shown in this study. As shown by Richard Baines [31], postsynaptic overexpression of a constitutively active form of PKA induces a change in the electrical properties of aCC, resulting in a decrease in excitability. Overexpression of a PKA inhibitor, on the other hand, does not modulate the excitability of aCC. In this study, we describe a perfect mirror image of this situation, namely that the expression of a PKA inhibitor induces a dendritic overgrowth phenotype, while expression of a constitutively active form of PKA does not. Bringing these two lines of observations together suggests the following model: following a decrease in presynaptic input (and PKA signalling), the postsynaptic neuron expands its receptive field so as to increase the number of presynaptic sites that it contacts. In these circumstances, an increase in postsynaptic excitability would not be required. On the other hand, following an increase in presynaptic input, the postsynaptic neuron decreases its excitability (via increased PKA signalling) without the need to reduce its receptive field.

We favour the view that electrical and structural homeostatic mechanisms might indeed be integrated by neurons. This would enable the cells to implement compensatory changes that resulted in adjustments to their electrical characteristics and dendritic geometry, so as to ensure that in an inherently variable environment an adequate pattern and level of connectivity and excitability is achieved.

## Materials and Methods

### Fly stocks.

Eggs were collected from flies kept on apple juice agar supplemented with yeast paste and maintained at 25 °C. Fly stocks and recombinant chromosomes were generated using standard procedures. Presynaptic terminals were labelled with *UAS-SytGFP* [28]. Expression of transgenes was targeted to cholinergic interneurons and excluded from aCC using the recombinant stock *w; Cha-Gal4, UAS-SytGFP; RN2Gal80* [18]. For controls, wild-type Oregon-R flies were crossed to this presynaptic driver stock. *UAS-TeTxLC* (*TNT-G*) was used to block evoked neurotransmitter release [25], *EP(3)381/ TM3, Sb, Kr::GFP* for the presynaptic expression of *faf* [30], and *UAS-Robo^ectodomain^Frazzled^cytoplasmic^* to shift the presynaptic terminals to a more medial region of the neuropile [32]. We used *Cha* ^L13^ */ TM3, Sb, Kr::GFP* flies to study the effect of lack of neurotransmitter [24], and the lethal allele of the acetylcholinesterase gene *Ace* ^J50^ / *TM3, Ser, act-GFP* to increase the exposure of postsynaptic neurons to acetylcholine. All balancer chromosomes were marked with green fluorescent protein (GFP) to allow for unambiguous identification of animals of the appropriate genotype, selected for using a Leica FLIII fluorescence stereo microscope. Gene expression was targeted to the postsynaptic aCC motor neurons (as well as the RP2 motor neuron and pCC interneuron, which does not synapse with motor neurons, M. Tripodi and M. Landgraf, unpublished data) with *RN2-GAL4* [38]. *UAS-PKAact^1^* was used to enhance PKA activity, and the dominant negative form of the PKA regulatory subunit, *PKAinh^1^*, to down-regulate it [37].

### Staging and injection of Lucifer Yellow.

Flies were left to lay eggs on agar plates for 12 h at 25 °C. Embryos were selected during the short window of time when their main dorsal tracheae begin to fill with air, which represents 18 h AEL. The VNC was dissected out in phosphate buffered saline (PBS, 0.075 M [pH 7.2]), fixed immediately in 4% paraformaldehyde (PFA in PBS) for 5 min at room temperature, and then washed several times in PBS. Injections of Lucifer Yellow were carried out as described previously [63].

### Antibody staining and image processing.

After Lucifer Yellow injection, a second fixation of 30 min in 4% PFA was applied, specimens were washed in PBS with 0.3% Triton-X, incubated with primary antibodies overnight at 25 °C in humidified chambers (rabbit anti-Lucifer Yellow 1:1,000, Invitrogen; mouse monoclonal antibodies [mAb] 1D4, anti-FasII 1:10; goat anti-GFP 1:1,500, AbCam), washed in PBS with 0.3% Triton-X, and incubated with Alexa488, Alexa568, and Alexa633 conjugated secondary antibodies (1:800, Invitrogen) for 2 h, washed in PBS Triton-X 0.3%. Specimens were cleared and mounted in Vectashield (Vector Laboratories) between two aluminium-foil spacers to avoid distortion of nerve cords under number 1 cover glasses. Images were collected using a Leica SP1 confocal laser scanning microscope. Confocal image stacks were imported and further processed with Amira 4.1.1. For reconstructions and quantifications of dendritic geometry and synaptic distribution, we used the software developed by Jan Felix Evers, described in [21–23].

### Statistical analysis.

For statistical analysis, ASCII-tables generated in Amira were imported in R and SPSS. Normally distributed data were analysed for statistical significance using two-tailed *t*-test for pairwise comparison, or using one-way ANOVA analysis for comparison of multiple datasets. For data whose distribution did not appear to be normal, the Wilcoxon rank-sum test was used (Figure 6). Unless otherwise stated, *p* < 0.05 is considered statistically significant.

## Supporting Information

Figure S1 Use of Genetically Encoded Presynaptic Markers (A–C) Single confocal sections of 18-h-AEL nerve cords stained with anti-GFP to visualize (A) the genetically encoded presynaptic marker synaptotagmin-GFP (pseudocoloured green) expressed in cholinergic neurons and (B) endogenous synapsin (red). (C) shows an overlay of the channels.
(D–F) As above, but showing staining for anti-synaptotagmin (endogenous and transgenic) in red (E). All synaptotagmin-GFP positive puncta colocalize with the endogenous presynaptic markers synapsin (C) and synaptotagmin (F).
In all the figures, HRP marks the neuropile and is pseudocoloured blue. Enlarged parts as indicated by the boxes are shown in the top left hand corners. Scale bars indicate 5 μm for main image and 1 μm for enlarged insets.
(1.08 MB PDF)

Figure S2 Nerve Cords of *Cha::TNT-G::myr-mRFP1* (Experimental; Active Tetanus Toxin) and *Cha::TNT-VIF::myr-mRFP1* (Controls; Inactive Tetanus Toxin) Animals Tetanus toxin was expressed in cholinergic neurons. Animals expressing *UAS-TNT-G* were identified based on the expression of the co-marker *UAS-myr-mRFP1*. DIC images are shown in the left column, the centre columns shows the expression of red fluorescent protein (RFP), and the right column shows the merge of the two channels.
Bottom row: analysis of the lengths of VNCs expressing active (TNT-G) and inactive (TNT-VIF) tetanus toxin in cholinergic neurons, respectively. The expression of the *TNT-G* in cholinergic neurons does not impair VNC condensation.
(1.34 MB PDF)

Figure S3 Expression of Chimeric Axon Guidance Receptors in the Cholinergic Neurons Does Not Obviously Affect FasII Axon Bundle Spacing (A) Staining of the FasII-positive fascicles in control nerve cords. (B) Staining of the FasII-positive fascicles in *Cha::Robo-Frazzled* animals.
(C) Quantification of the interfascicle distances for control animals (red) and *Cha::Robo-Frazzled* animals (cyan). Distances were measured between the lateral and the intermediate fascicles, the lateral and the medial fascicles, and the medial fascicle and the midline. The expression of the chimeric receptor *Robo-Frazzled* does not alter the spacing of FasII fascicles, suggesting that the Slit gradient is not affected by this manipulation. I, Intermediate; L, Lateral FasII-positive fascicle; M, Medial; mid, midline. The *y*-axis indicates distance in micrometres, the *x*-axis interfascicle distance in micrometres; *n* = 10.
(1.07 MB PDF)

## Abbreviations

*Ace,*: acetyl choline esterase
AEL: after egg laying
*Cha,*: choline acetyl transferase
Faf: fat-facets
FasII: fasciclin II
PKA: protein kinase A
TNT: tetanus toxin
VNC: ventral nerve cord
