# Supplementary figures and images for: Structural Homeostasis: Compensatory Adjustments of Dendritic Arbor Geometry in Response to Variations of Synaptic Input
Source: PLoS Biol. 2008 Oct 28;6(10):e260. doi: 10.1371/journal.pbio.0060260 (PMC2573934; doi:10.1371/journal.pbio.0060260)

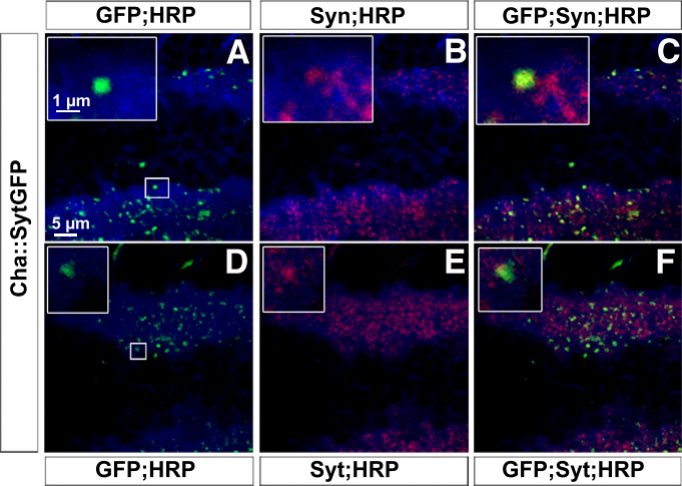

Supplement: Figure S1 — (A–C) Single confocal sections of 18-h-AEL nerve cords stained with anti-GFP to visualize (A) the genetically encoded presynaptic marker synaptotagmin-GFP (pseudocoloured green) expressed in cholinergic neurons and (B) endogenous synapsin (red). (C) shows an overlay of the channels. (D–F) As above, but showing staining for anti-synaptotagmin (endogenous and transgenic) in red (E). All synaptotagmin-GFP positive puncta colocalize with the endogenous presynaptic markers synapsin (C) and synaptotagmin (F). In all the figures, HRP marks the neuropile and is pseudocoloured blue. Enlarged parts as indicated by the boxes are shown in the top left hand corners. Scale bars indicate 5 μm for main image and 1 μm for enlarged insets. (1.08 MB PDF) [file pbio.0060260.sg001.pdf]

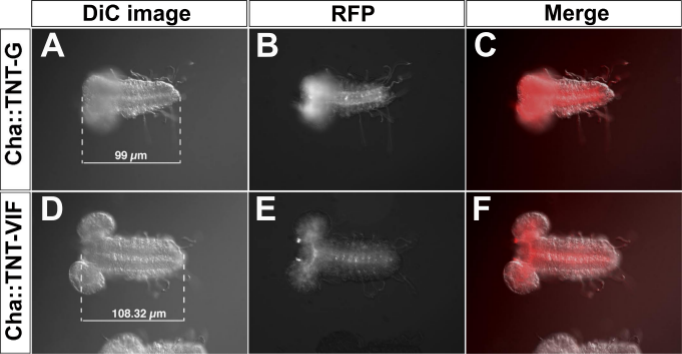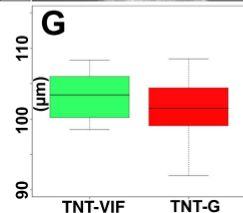

Supplement: Figure S2 — Tetanus toxin was expressed in cholinergic neurons. Animals expressing UAS-TNT-G were identified based on the expression of the co-marker UAS-myr-mRFP1. DIC images are shown in the left column, the centre columns shows the expression of red fluorescent protein (RFP), and the right column shows the merge of the two channels. Bottom row: analysis of the lengths of VNCs expressing active (TNT-G) and inactive (TNT-VIF) tetanus toxin in cholinergic neurons, respectively. The expression of the TNT-G in cholinergic neurons does not impair VNC condensation. (1.34 MB PDF) [file pbio.0060260.sg002.pdf]

Control

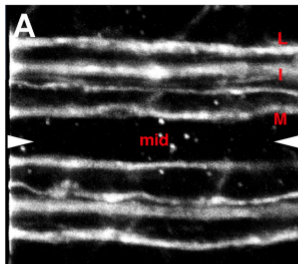

Cha::Ro-Fra

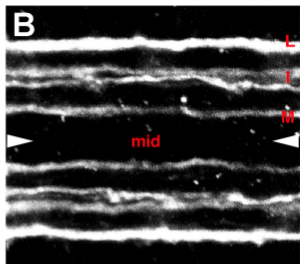

FasII distance

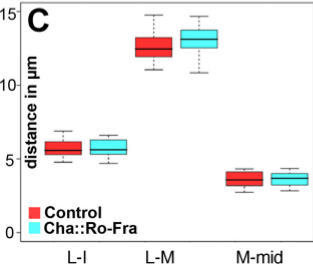

Supplement: Figure S3 — (B) Staining of the FasII-positive fascicles in Cha::Robo-Frazzled animals. (C) Quantification of the interfascicle distances for control animals (red) and Cha::Robo-Frazzled animals (cyan). Distances were measured between the lateral and the intermediate fascicles, the lateral and the medial fascicles, and the medial fascicle and the midline. The expression of the chimeric receptor Robo-Frazzled does not alter the spacing of FasII fascicles, suggesting that the Slit gradient is not affected by this manipulation. I, Intermediate; L, Lateral FasII-positive fascicle; M, Medial; mid, midline. The y-axis indicates distance in micrometres, the x-axis interfascicle distance in micrometres; n = 10. (1.07 MB PDF) [file pbio.0060260.sg003.pdf]
